# Mapping the evidence on virtual reality for dementia caregivers: a scoping review

**DOI:** 10.1093/her/cyag003

**Published:** 2026-02-11

**Authors:** Xiaoli Li, Qiwei Li, Kang Sun, Reagen Conklin

**Affiliations:** School of Health Sciences, Southern Illinois University Carbondale, 1365 Douglas Drive Carbondale, IL 62901-6604, United States; Department of Public Health, California State University, Fresno, 2345 East San Ramon Avenue M/S MH30 Fresno, CA 93740, United States; School of Social Work Southern Illinois University Carbondale, 1365 Douglas Drive Carbondale, IL 62901-6604, United States; School of Health Sciences, Southern Illinois University Carbondale, 1365 Douglas Drive Carbondale, IL 62901-6604, United States

## Abstract

Virtual reality (VR) is increasingly recognized as a promising tool for healthcare training, yet its application in dementia caregiver education remains an emerging field. Despite growing interest, few reviews have systematically mapped how VR interventions are used to support both formal and informal caregivers. This scoping review aims to synthesize current evidence on VR-based dementia care training and to identify how VR contributes to caregiver knowledge, skills, empathy, and preparedness. Seventeen studies were included, encompassing diverse caregiver populations such as family caregivers, certified nursing assistants, nurses, and other healthcare professionals, with sample sizes ranging from 10 to 223 participants. Study designs varied and included pre–post studies (*n* = 3), randomized controlled trials (*n* = 3), non-randomized controlled trials (*n* = 1), feasibility pilot studies (*n* = 1), quasi-experimental designs (*n* = 4), qualitative studies (*n* = 3), and post-test-only designs (*n* = 2). Research was conducted across the United States, Ireland, Australia, South Korea, Taiwan, and the United Kingdom. VR interventions included the Virtual Dementia Tour, Into D’mentia, Through the D’ementia Lens, 360° VR films, and A Walk-Through Dementia, reflecting substantial variation in content and delivery formats. Overall, the evidence indicates that VR is an engaging and impactful educational modality that enhances caregivers’ empathy, dementia-related knowledge, and care competence. These findings highlight VR’s potential as a valuable complement to traditional dementia care training. However, methodological variability, limited long-term follow-up, and inconsistent outcome measures underscore the need for more rigorous and standardized research to inform best practices and guide implementation across diverse caregiving contexts.

## Introduction

The World Alzheimer revealed that ~57 million people worldwide were affected by Alzheimer’s Disease and Related Dementias (ADRD) [1], with 6.9 million residing in the USA alone in 2024 [2]. With dementia cases continuing to rise, projections indicate that the number of people with ADRD in the USA will increase 1.8 million between 2023 and 2030 [3, 4]. According to the World Health Organization, dementia is the seventh leading cause of death globally, thus researching this disease, as well as factors that can improve the quality of life for dementia patients and enhance dementia care training for caregivers, is crucial [2].

Dementia, a progressive disease that worsens over time, is primarily characterized by cognitive deterioration. It manifests in various forms, including Alzheimer’s disease (which accounts for 60%–70% of dementia cases), vascular dementia, and Lewy body dementia [5]. Although not all forms of dementia fall under this category, ADRD accounts for a substantial proportion of dementia cases. Individuals affected by ADRD often experience symptoms such as memory loss, impaired decision-making, and other cognitive deficits, such as apraxia (unable to do things), amnesia (forgetfulness), aphasia (unable to talk well), and agnosia (unable to recognize) [2].

Given the severity of these symptoms, it is crucial for caregivers to approach dementia care with understanding and empathy [3]. Dementia care training encompasses programmes, training, and other resources designed to enhance caregivers’ empathy, knowledge, and skillsets when working with individuals with ADRD. The National Council of Certified Dementia Practitioners highlights multiple benefits of effective dementia care training. More specifically, it enhances caregivers’ knowledge in long-term care facilities [6], but it also improves communication between caregivers and patients, strengthens behavioural management skills, and therefore reduces dementia-related behaviours such as aggression, psychosis, agitation, and other symptoms [7, 8]. However, due to insufficient caregiver training, pharmacologic interventions often remain the primary method to manage these behaviours. Implementing comprehensive dementia care training could lead to non-pharmacological, behavioural management strategies and an improved quality of life for patients with ADRD.

Dementia training for caregivers of individuals with ADRD can vary, ranging from educational PowerPoint presentations to advanced technology-based approaches. Traditional training methods, such as classroom-based education, have been widely used to enhance dementia care knowledge among caregivers in the long-term care field. Attending lectures designed to deepen understanding of dementia can help caregivers recognize and respond appropriately to various aspects of the disease. While studies have shown improvements in learning, they suggest that these methods do not lead to significant changes in dementia care practices [9, 10]. Interprofessional education (IPE), an approach in which professionals from two or more disciplines learn together, can also play a significant role in dementia training [10]. When dementia care providers engage in IPE, it increases educational collaboration and improved ability to manage dementia care more effectively [11]. In addition to educational lectures, training programmes focused on behavioural understanding and management are commonly implemented. Gitlin *et al.* [12] examined the effectiveness of the ‘Advancing Caregiver Training’ (ACT) programme in managing behavioural symptoms associated with dementia. ACT is a 16-week course based on the idea that ‘problem behaviours’ can arise from the domains of patient-based, caregiver-based, and environment-based stresses [12]. These types of training programmes have existed for decades, with organizations such as the Alzheimer’s Association continuously developing new training initiatives to further improve dementia care.

In addition to traditional learning and training, technology is increasingly being used in dementia care training for caregivers. Muirhead *et al.* [13] conducted a systematic review of the technological characteristics associated with effective dementia care training. The review identified several technological training methods, including internet-based learning, simulations, and tele-mentoring. Research conducted by Surr *et al.* [14] found that online training programmes incorporating interactive elements such as images, videos, audio, and graphics were more effective than passive methods. The other studies on technology-based education were effective in improving caregiver confidence, knowledge, and skills due to their flexibility, accessibility, and ability to reach individuals in diverse locations [13, 15].

Furthermore, the use of virtual reality (VR) as an educational tool has gained significant attention due to its potential to provide a more engaging and hands-on learning experience. VR is defined as a 3D, computer-generated environment that users can explore and interact with [16]. In dementia care training, VR can be used to simulate the perspective of someone living with dementia, offering an immersive experience that closely mirrors real-world challenges [17]. This technology has shown potential as an effective strategy for increasing caregiver empathy and knowledge in dementia care [18]. In a qualitative study, researchers used structured interviews and questionnaires to assess the effectiveness of VR training for dementia awareness. They found that 85% of caregivers considered VR training beneficial, while 76% reported that it influenced their caregiving approach over the course of a longitudinal study [19]. Similarly, Tsai *et al.* [20] implemented the VR training programme Through the D’ementia Lens with 35 caregivers of individuals with ADRD, finding that most participants reported an increased understanding of their patients’ perspectives. In another study, Slater *et al.* [21] evaluated the Virtual Dementia Tour (VDT) with 223 healthcare professionals, observing significant improvements in knowledge and understanding from pretest to post-test (*P* < .01) across all 15 questionnaire items. Additionally, Hicks *et al.* [17] utilized the VR training programme A Walk-Through Dementia with home care practitioners supporting individuals with ADRD. Through focus groups, caregivers described the VR training as providing ‘next level’ dementia care awareness. By offering an immersive experience, VR training has demonstrated greater effectiveness in enhancing caregiver understanding compared to traditional training methods.

Although VR has gained increasing attention as a tool for caregiver education, existing studies vary widely in their intervention designs, targeted caregiver populations, and outcome measures. Many of these studies have examined only limited caregiver groups, focusing primarily on nursing students, professional care staff, or single-programme evaluations. As a result, the broader landscape of VR applications across the full spectrum of dementia caregivers remains unclear. This scoping review addresses this gap by systematically synthesizing studies involving both formal caregivers (e.g. nurses, certified nursing assistants, long-term care staff, allied health professionals) and informal caregivers (e.g. family caregivers, home-based caregivers). By integrating evidence across these groups, the review provides a more comprehensive understanding of how VR functions as an educational tool in diverse caregiving contexts, identifies the types of VR interventions implemented, summarizes the caregiver outcomes reported in the literature, and highlights key methodological limitations that warrant further investigation.

A scoping review methodology was selected because research on VR-based dementia caregiver training is still in an early, exploratory stage and characterized by substantial heterogeneity in intervention design, caregiver populations, and outcome measures. In accordance with Arksey and O’Malley’s framework [22] and subsequent guidance from Levac *et al.* [23] and the Joanna Briggs Institute (JBI), a scoping review is appropriate when the goal is to map the breadth of existing evidence, clarify key concepts, and identify gaps in an emerging field rather than evaluate intervention effectiveness. Given the variability and limited number of existing studies, a systematic review would not be feasible or meaningful at this stage, and a narrative review would not provide the methodological transparency necessary for reproducibility. Therefore, a scoping review offers the most suitable approach to comprehensively synthesize current knowledge and guide future research.

## Methods

This scoping review was conducted to examine studies on the use and effectiveness of VR in dementia care training for caregivers of individuals with ADRD, following the methodological framework proposed by Arksey and O’Malley [22], enhanced by Levac *et al.* [23], and guided by the JBI Manual for Evidence Synthesis [24]. The review followed these key steps: (i) defining the research question, (ii) identifying and selecting relevant studies using predefined inclusion and exclusion criteria, (iii) extracting and organizing data into structured tables, and (iv) synthesizing the findings to address the research question. Reporting was conducted in accordance with the Preferred Reporting Items for Systematic Reviews and Meta-Analyses extension for Scoping Reviews (PRISMA-ScR) checklist [25] to ensure methodological transparency and reproducibility. The PRISMA-ScR checklist guided the documentation of the search strategy, study selection, data extraction, and synthesis processes.

### Identifying the research question

The following research questions guided this scoping review:

RQ1: What types of VR interventions have been implemented for dementia care training?

RQ2: What caregiver outcomes have been examined in existing studies, and how have these outcomes been reported?

RQ3: What methodological gaps and limitations exist within current research on VR-based dementia caregiver training?

These questions were designed to systematically map the extent, range, and nature of research on VR in dementia care training, summarize the outcomes assessed, and identify gaps and areas for future investigation, rather than evaluate the effectiveness of VR interventions.

### Identifying relevant studies

This review included studies published in English that examined the role of VR interventions in dementia care training using quantitative, qualitative, or mixed methods approaches. A systematic search strategy was developed to identify relevant studies. Search terms were selected to capture three key concepts: (i) VR interventions, (ii) dementia care training, and (iii) caregiver populations. Terms for VR included ‘virtual reality,’ ‘VR,’ ‘simulated reality,’ ‘360 video,’ and ‘3D video.’ Terms for dementia care training included ‘dementia care training,’ ‘Alzheimer disease care training,’ and ‘Alzheimer disease–related dementia education.’ Caregiver-related terms included ‘caregiver,’ ‘informal caregiver,’ ‘care worker,’ ‘certified nurse assistant,’ and ‘professional caregiver.’ Both informal caregivers (e.g. family members, part-time caregivers) and formal caregivers (e.g. nurses, certified nursing assistants, long-term care staff) were included because VR interventions may target either or both groups across diverse care settings (see Table 1). Search terms were applied across six databases: Academic Search Complete, APA PsycINFO, CINAHL, MEDLINE, PubMed, and Web of Science. Because each database has unique filtering characteristics, the search strategy was tailored for each to ensure retrieval of the most relevant studies. Additionally, Google Scholar and citation tracking were used to supplement database searches.

**Table 1:** Database search terms.

Search terms	Notes
‘VR’ OR ‘Virtual Reality’ OR ‘Simulated Reality’ OR ‘360 video’ OR ‘3D video’	Captures all forms of VR-based interventions
‘Dementia care training’ OR ‘Alzheimer disease care training’ OR ‘Alzheimer disease–related dementia education’	Focus on education programmes for dementia
‘Caregiver’ OR ‘Informal caregiver’ OR ‘Family caregiver’ OR ‘Care worker’ OR ‘Certified nurse assistant’ OR ‘Professional caregiver’	Includes both informal (family, part-time) and formal caregivers (nurses, staff)

### Selecting studies

Studies were included if they: (i) examined VR interventions specifically designed for dementia care training, (ii) involved caregivers of individuals with dementia, including informal caregivers (e.g. family members, part-time caregivers) and formal caregivers (e.g. nurses, certified nursing assistants, and professional caregivers), (iii) employed quantitative, qualitative, or mixed-methods designs, and (iv) were published in English between 2000 and 2025. Studies were excluded if they were reviews, commentaries, conference abstracts, protocols, or non-English publications (Table 2). The timeframe was selected to capture studies using VR technology relevant to current dementia care training practices, as VR applications in healthcare training have evolved substantially since the early 2000s [26]. These criteria were developed to ensure that included studies were directly relevant to the scope of this review while maintaining methodological rigour and focus.

**Table 2:** Article inclusion criteria.

Criteria	Inclusion	Exclusion
Participants	Formal caregivers (e.g. nurses, certified nursing assistants, professional care staff) and informal caregivers (e.g. family members, part-time caregivers)	Populations not involved in dementia caregiving
Intervention	VR interventions specifically designed for dementia care training	Non-VR interventions or studies not focused on dementia care training
Study Design	Quantitative, qualitative, or mixed-methods studies	Reviews, commentaries, conference abstracts, protocols
Language	Published in English	Non-English publications
Publication Date	2000–25	Published before 2000

### Data extraction

Data was organized into structured charts to systematically analyse the key characteristics of each included study [27]. For all selected articles, the following information was extracted by two researchers (X.L. and R.C.) independently, with cross-checking conducted to ensure accuracy: author(s), year of publication, participant characteristics, study design, measurement tools, key findings, care settings, and the type of VR intervention implemented. This structured extraction process allowed us to identify the types of VR interventions used in dementia care training and to summarize the reported outcomes associated with these interventions.

## Results

### Studies identified

The study selection process began in July 2024. The initial database search using terms related to VR, dementia caregiving, and caregiver training yielded 45 records across the six selected databases. After removing 5 duplicates, 40 unique records were screened by two independent reviewers (X.L. and R.C.) based on titles and abstracts. During this stage, 16 records were excluded for not meeting the inclusion criteria (e.g. not involving VR, not related to dementia care training, or not including caregiver participants).

The remaining 24 full-text articles were assessed independently by the same two reviewers (X.L. and R.C.). Reasons for exclusion at this stage were documented, including article type (e.g. reviews, commentaries, or reports; *n* = 17) and lack of full-text availability or non-English language (*n* = 2). Discrepancies between reviewers were resolved through discussion with other two reviewers (Q.L. and K.S.). Ultimately, 5 studies retrieved from the database search met all inclusion criteria and were included in the review [1, 22–25].

In the second stage, 236 records were identified through Google Scholar and 12 additional records through citation searching. After removing 4 duplicates, 244 records underwent title and abstract screening by two independent reviewers (X.L. and R.C.). A total of 142 records were excluded for irrelevance to VR dementia training. The remaining 102 full-text articles were evaluated for eligibility. Of these, 66 were excluded because they were reviews, commentaries, or conference papers, and 24 were excluded due to lack of full-text availability. Again, discrepancies were resolved through discussion with other two reviewers (Q.L. and K.S.). After full-text assessment, 12 studies met the inclusion criteria and were added to the review [4, 17–19, 21, 28–34].

In total, 17 studies were included in the final synthesis. A PRISMA-ScR flow diagram (Fig. 1) provides a visual summary of the selection process, including reasons for exclusion at each stage.

**Figure 1:**
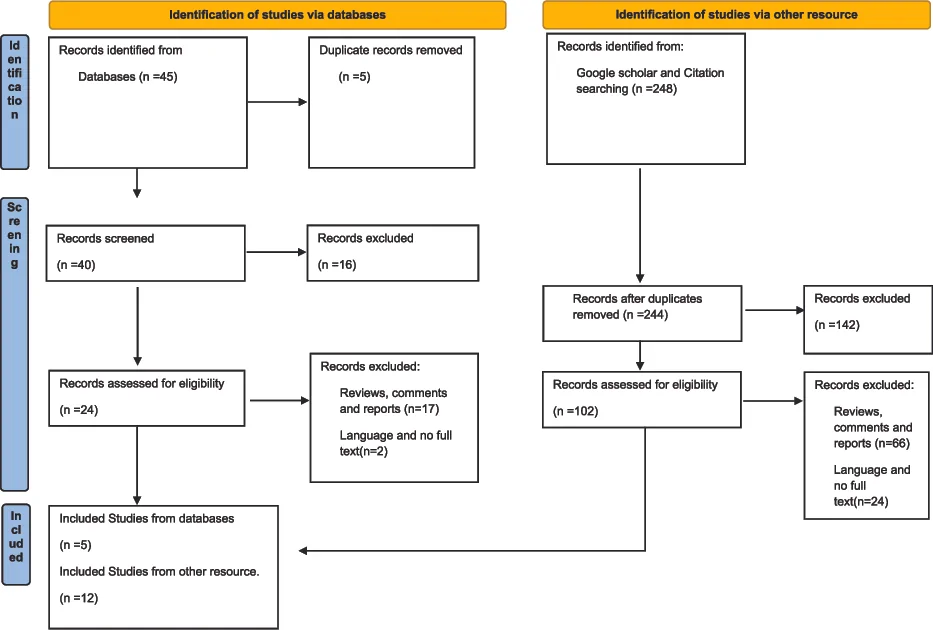
The PRISMA-ScR flow diagram of study selection process.

### Key characteristics of studies

Using the 17 selected studies, Table 3 was created to summarize the measurements, key findings, VR interventions, and other main characteristics of studies investigating VR in dementia care training. The studies included a diverse range of participants, such as caregivers (family or part-time caregivers), nurses, certified nursing assistants, and other healthcare professionals working with dementia patients, with sample sizes ranging from 10 to 223 participants. Study designs varied, including pre-test-post-test designs (three studies), randomized controlled trials (three studies), non-randomized control trials (one study), feasibility pilot studies (one study), quasi-experimental designs (four studies), qualitative studies (three studies), and post-test-only designs (two studies).

**Table 3:** The summary of included studies.

(Author, year, country)	Sample/study design	Instrument/measurement	Key findings	Settings	VR intervention
Aicken *et al.*, 2021 (UK)	Qualitative; 24 health care assistants	Semi-structured interviews; feedback forms	VR increased understanding among caregivers of people with dementia.	Two UK nursing homes	A walk-through dementia
Hicks *et al.*, 2023 (UK)	Qualitative; 20 care home practitioners	Semi-structured focus groups	VR experience was immersive, insightful, and emotionally engaging.	Four UK care homes	A walk-through dementia
Gómez-Morales *et al.*, 2024 (USA)	Pre–post pilot; 20 family caregivers	Zoom interviews	Improved understanding of memory loss, confidence, and perceived caregiving ability.	Phoenix, Arizona	Through Alzheimer’s eyes
Jutten *et al.*, 2018 (Netherlands)	Quasi-experimental longitudinal; 145 intervention, 56 control	Interpersonal Reactivity Index, Caregiver Reaction Assessment, Hospital anxiety and depression scale, Relationship Quality Index	VR was useful; increased understanding; no significant group differences.	Netherlands	Into D’mentia
Kim, 2021 (Korea)	Methodological; 85 caregivers	Questionnaire	Increased caregiver confidence in dementia care.	College of Nursing, Korea	VR practice programme
Morganti *et al.*, 2024 (Italy)	Quasi-experimental; 36 informal caregivers	State–Trait Anxiety Inventory, Beck Depression Inventory, Zarit Burden Inventory, Interpersonal Reactivity Index	Higher presence among those with higher empathy, age, and lower distress.	Memory Clinic, Brescia	ViveDe video scenarios
O’Connor *et al.*, 2014 (USA)	Feasibility pilot; 30 informal caregivers	UCLA Loneliness Scale, Geriatric Depression Scale, Perceived Stress Scale	Stress approached significance; small improvements in mood and loneliness.	Tucson, Arizona	Second life virtual environment
Slater *et al.*, 2019 (Ireland)	Qualitative; 52 caregivers	Interviews	VR training useful; increased empathy.	Irish health service settings	VDT
Slater, 2021 (Ireland)	Quasi-experimental; 223 professional carers	Pre–post questionnaire	Significant increases in empathy and understanding.	Northern Ireland Health & Social Care Trust	VDT
Solecki *et al.*, 2021 (USA)	Quasi-experimental pre–post; 56 nursing staff	Pre/post survey, Perception of Caring Survey	Slight improvement in attitudes and optimism.	Midwest medical–surgical units	VDT
Stargatt *et al.*, 2021 (Australia)	RCT; 60 VR, 54 control	Situational Empathy & Perspective Taking Scale, Dementia Knowledge Assessment Scale, Dementia Attitudes Scale	VR improved knowledge and attitudes; mixed empathy results.	Australia	Educational dementia immersive experience
Stargatt *et al.*, 2022 (Australia)	RCT; 40 VR, 56 control	Understanding Dementia Care Environments	Improved understanding of dementia-friendly environmental modifications.	Residential aged care facilities	VR tour
Sung *et al.*, 2022 (Taiwan)	Cluster RCT; 61 VR, 63 control	Dementia Knowledge Assessment Scale, Approaches to Dementia Questionnaire, Jefferson Scale of Empathy	Significant gains in knowledge, attitudes, empathy, competence.	Long-term care settings	VR-based activity
Tsai *et al.*, 2023 (Taiwan)	Post-test; 10 formal caregivers	System usability scale for VR, VR experience appraisal scale	SUS score 74.06; increased dementia understanding.	Long-term care institution	Oculus VR videos
Wijma *et al.*, 2018 (Netherlands)	Pre–post; 35 informal caregivers	Questionnaire (empathy, competence, relationship quality)	Participants gained insight; VR rated 8.03/10.	Four Dutch care institutes	Through the D’ementia lens
Wu *et al.*, 2024 (Japan)	Pre–post; 76 nurses	Attitudes Toward Dementia Scale, Vignettes, Self-Efficacy Scale	Improved attitudes and confidence (41.9 → 44.5).	Tokyo General Hospital	70-min VR movies
Yamaguchi *et al.*, 2022 (Japan)	Non-randomized control trial; 39 acute care nurses	Dementia Nursing Competency Scale, Dementia Attitude Scale, Knowledge in Dementia Scale	Significant improvements in competency and staff experiences.	Kobe University Hospital	Oculus go VR scenarios

The studies were conducted in various countries and regions, including the United States, Ireland, Australia, South Korea, Taiwan, and the United Kingdom, indicating widespread interest in VR-based dementia training. The VR interventions included in the reviewed studies encompassed a range of immersive programmes designed to enhance caregiver understanding and skills. VDT simulates the physical and cognitive impairments experienced by individuals with dementia, allowing caregivers to gain empathy by experiencing memory loss, sensory limitations, and disorientation. Walk-Through Dementia and Into D’mentia provide first-person perspectives of daily life for people with dementia, highlighting challenges in performing routine tasks and navigating environments. Through the D’ementia Lens emphasizes perspective-taking by simulating the sensory, cognitive, and emotional effects of dementia in realistic scenarios. 360° VR videos offer panoramic views of caregiving situations to enhance situational awareness, while custom immersive scenarios allow caregivers to practice communication strategies, person-centred care, and responses to behavioural and psychological symptoms. These VR applications aim to foster empathy, improve knowledge, and enhance competence in dementia care across both formal and informal caregiver groups.

### Thematic findings: applications and effects of VR in dementia care training

Synthesis of the 17 included studies revealed three major thematic areas. First, the content and application of VR interventions focused on simulating the sensory, cognitive, and emotional challenges experienced by individuals with dementia. These VR experiences helped caregivers better understand visual and auditory distortions, behavioural and psychological symptoms, and the environmental factors that contribute to confusion or distress [17, 21]. The interventions often aimed to cultivate empathy, enhance perspective-taking, support person-centred care strategies, and improve communication skills [18, 35]. VR programmes were delivered across diverse settings, including long-term care facilities, nursing education programmes, dementia workshops, and home-based caregiver support programmes, highlighting their adaptability for both formal and informal caregivers [4, 17, 21].

Second, studies employed a wide range of outcome measures to evaluate VR-based training. Quantitative investigations used validated tools to assess empathy (e.g. Jefferson Scale of Empathy, Emotional Attunement Questionnaire) [18, 32], dementia knowledge (e.g. Dementia Knowledge Assessment, Approaches to Dementia Questionnaire) [18, 36], attitudes toward dementia (e.g. Dementia Attitudes Scale) [34, 36], care competence (e.g. Sense of Competence in Dementia Care Staff Scale) [18], and caregiver stress or emotional response (e.g. Perceived Stress Scale) [33]. Usability assessments were also reported, such as a System Usability Scale score of 74.06 in Tsai *et al.* [1]. Qualitative studies complemented these measures by using interviews, open-ended surveys, and focus groups, which often emphasized the realism, emotional resonance, and practical relevance of VR experiences [17, 31].

Third, reported effects of VR interventions were generally positive. Most studies found that VR increased caregiver empathy, improved dementia knowledge and care strategies, and enhanced confidence and competence in person-centred care [18, 35, 36]. Caregivers frequently described gaining emotional insight into the lived experience of dementia, including how sensory or environmental factors may trigger behavioural expressions [17, 21]. However, two studies found no statistically significant improvements following VR training [19, 36], with authors suggesting that user characteristics, such as previous caregiving experience, familiarity with technology, or learning preferences, may influence outcomes. Overall, the evidence positions VR as a promising tool for dementia care training, while underscoring the need for standardized outcome measures, larger and more diverse samples, and longitudinal follow-up to strengthen the evidence base.

## Discussion

This review summarizes the current research on VR interventions used in dementia care training for both formal caregivers (e.g. nurses, nursing assistants, health professionals) and informal caregivers (e.g. family members, home-based caregivers). By synthesizing 17 studies, we identified how VR is being applied, which outcomes are being measured, and how these interventions influence dementia-related knowledge, empathy, competence, and attitudes. While our findings align with previous reviews that highlight the potential of VR to enhance caregiver preparedness and person-centred care skills [37, 38], this scoping review extends the existing literature in several important ways.

First, unlike earlier reviews that focused primarily on single caregiver groups (e.g. nursing students or professional staff) [39, 40], or on specific VR programmes [41], our review integrates evidence across both formal and informal caregivers, providing a more comprehensive picture of how VR is used in diverse caregiving contexts. Second, we include more recent studies published through 2025, capturing newly developed VR applications and updated methodological approaches that were not examined in prior reviews [42]. This is particularly meaningful in VR studies as VR itself evolves rapidly, and a few years gap could lead to significant changes in content generation, reality simulation, and outcomes. Third, this review offers a deeper analysis of the types of outcomes measured, the spectrum of VR interventions, and thematic applications (e.g. empathy building, sensory distortion simulation, communication training), providing a clearer mapping of how VR functions as an educational tool.

VR training showcases incredible user acceptance. Most caregivers, including nurses, health care assistants, and informal family caregivers, reported positive experiences with VR as an educational tool. For instance, VR interventions were described as immersive and insightful, significantly increasing dementia awareness among care home practitioners [17]. Likewise, Jutten *et al.* found that the majority of participants considered VR training useful and believed it enhanced their understanding of dementia [19]. Slater *et al.* [21] also observed significant improvements across demographic groups in empathy and understanding following a structured VR education programme. Collectively, these findings suggest that VR can effectively bridge the gap between theoretical knowledge and practical understanding by enabling caregivers to experience sensory, perceptual, and behavioural challenges from the perspective of a person living with dementia.

However, the effectiveness of VR was not universal across all participants or outcomes. Informal caregivers reported gains in empathy, emotional understanding, and confidence, domains directly related to their caregiving relationships. In contrast, formal caregivers, particularly nurses and nursing assistants, demonstrated improvements in dementia knowledge, care competence, and person-centred care practices [18, 34, 43]. Stargatt *et al.* [36] found that improvements in empathy, knowledge, and attitudes varied depending on participant demographics, particularly age, indicating that VR may resonate differently with distinct learner profiles. This highlights that VR’s impact is influenced by factors such as prior caregiving experience, comfort with technology, learning preferences, or age-related differences in perceptual processing. These variations underscore the importance of tailoring VR training to specific caregiver groups rather than assuming uniform effectiveness. VR itself as a tool does not guarantee educational enhancement. The content of the training and its fit for the targeted audience play a more significant role. This indicates that the training design should be content-centred instead of equipment-centred.

Although results across studies were promising, VR research in dementia care training remains in an early developmental stage. Several gaps were identified. First, many studies lacked long-term follow-up; Hicks *et al.* [17] acknowledged that their study lacked systematic assessment of sustained effects, while Gómez-Morales *et al.* [4] noted the limited duration of their intervention. Slater *et al.* [21] also emphasized the importance of long-term evaluation to determine whether VR-induced improvements persist. Second, methodological variability, including diverse measurement instruments, inconsistent study designs, and non-standardized intervention protocols, limits comparability across studies and restricts conclusions about the general effectiveness of VR. Third, important practical barriers remain underexplored. VR sickness symptoms were noted as a concern in some studies [1, 29], and issues related to accessibility and financial burden were highlighted by Slater *et al.* [21] and Hicks *et al.* [17]. These considerations are critical for assessing the feasibility of integrating VR into routine dementia care education.

As global dementia prevalence rises, identifying effective caregiver training strategies is increasingly urgent. VR offers a unique contribution by providing experiential learning opportunities that traditional didactic methods cannot replicate. By fostering empathy, enhancing dementia knowledge, and improving perceived competence, VR has the potential to enrich dementia care practice and support higher-quality, person-centred care for individuals living with dementias. However, to advance the field, more rigorous and longitudinal research is needed to clarify the mechanisms through which VR exerts its effects, identify which caregiver groups benefit most, and determine how VR can be implemented sustainably and equitably across diverse care settings.

### Limitations

This scoping review has several limitations that should be considered when interpreting the findings. First, consistent with established scoping review methodology outlined by the JBI, we did not conduct a formal critical appraisal of the methodological quality of included studies, which limits our ability to draw conclusions about the strength or reliability of the evidence. Second, the review included only studies published in English. As a result, potentially relevant research published in other languages may have been missed, which could narrow the scope of the evidence base and limit global representation. Third, substantial methodological heterogeneity across studies made it difficult to compare findings directly or synthesize outcomes in a standardized manner. This diversity reflects the early and evolving nature of VR-based dementia caregiver training, but also restricts the ability to identify consistent patterns across studies. Finally, the rapidly evolving nature of VR technology means that some included interventions may already be outdated, and more recent innovations may not yet be reflected in the published literature.

These limitations suggest that while the findings offer a comprehensive overview of the current landscape, caution should be exercised when interpreting the conclusions. Future research using standardized methodologies, formal quality assessment, and inclusion of multilingual and grey literature will help strengthen the evidence base.

## Conclusion

Dementia care education is a critical area of research and practice, particularly given the growing number of individuals living with dementia worldwide. Although the included studies employed diverse methods, measured different caregiver characteristics, and used varied VR technologies, the overall findings indicate that VR is a promising and impactful tool for dementia care training. The existing evidence suggests that VR can enhance caregiver empathy, knowledge, competence, and understanding of dementia. Future research should prioritize standardized methodologies, longitudinal designs, and exploration of VR’s effectiveness across diverse caregiving roles and settings. Addressing practical barriers related to cost, accessibility, and implementation feasibility will be essential to ensure that VR-based training can be widely adopted and sustainably integrated into dementia care education.

## Data Availability

The datasets generated during and/or analysed during the current study are available from the corresponding author on request.
